# Effects of temperature and humidity on the performance of a PECH polymer coated SAW sensor

**DOI:** 10.1039/d0ra02502j

**Published:** 2020-05-11

**Authors:** Yong Pan, Lin Zhang, Bingqing Cao, Xufeng Xue, Weiwei Liu, Caihong Zhang, Wen Wang

**Affiliations:** State Key Laboratory of NBC Protection for Civilian Beijing 102205 China panyong71@sina.com.cn; Institute of Acoustics, Chinese Academy of Sciences Beijing 100190 China; School of Chemistry and Chemical Engineering, Shanxi University Taiyuan 030006 China

## Abstract

The influences of environment, such as temperature, humidity and interfering gases, on the performance of a surface acoustic wave (SAW) sensor in the detection of 2-chloroethyl ethyl sulfide (CEES) were invested. The 150 MHz SAW dual delay lines were used, coated with a poly(epichlorohydrin) (PECH) thin layer, and CEES was detected under different concentrations. Linear correlation between the frequency-shift and the exposure time of the sensor to CEES could be observed, and the limit of CEES could be detected as low as 1.5 mg m^−3^. Under different temperature (0–50 C°) and humidity (30–80% RH) conditions, CEES was detected by the fabricated SAW sensor coated with PECH, the frequency shifts were measured and the performance of the sensor was evaluated. The results proved that temperature and humidity were the most important factors to influence the performance of SAW sensors; with the decreasing of temperature and the increasing of humidity, there would be larger frequency shifts. In the interference experiments, it was found that most gases existing in the environment in high concentrations would not influence the detection of CEES. Then, the SAW sensor having been fabricated was kept under the conditions of 25 °C and 35% RH for 18 months to further verify the quality, and CEES was detected every so many months. It proved that the performance of the sensor would decrease about 16.39% after 18 months. Although it reflected the attenuation of the sensor to some extent, the sensor was still in good condition. Additionally, the related mechanisms were also discussed.

## Abstract 2

The influences of environment, such as temperature, humidity and interfering gases, on the performance of a surface acoustic wave (SAW) sensor in the detection of 2-chloroethyl ethyl sulfide (CEES) were invested.

## Graphical abstract

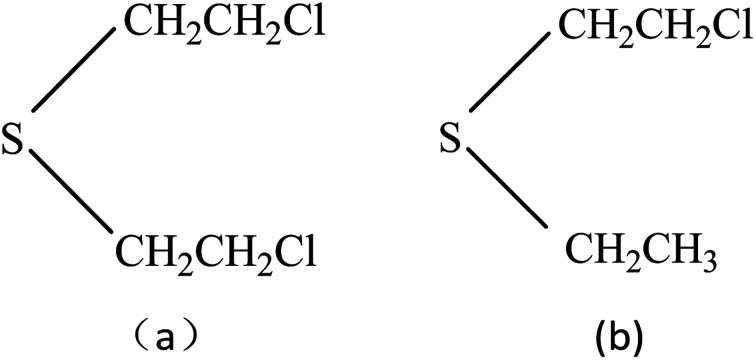

## Introduction

Chemical warfare agents (CWAs) are powerful weapons and a threat for the world; these agents still remain a threat especially from some countries and terrorists as these CWAs are easy to manufacture and have devastating effects. Although the possibility of occurrence is relatively small, the CWAs may still cause great harm to humans and the environment. The periodic use of chemical weapons in history for war and terror has created a need for the development of rapid detection and sensitive analytical methods and instrumentation. As a result, more and more people are working to detect them at very low limits using a large number of technologies, and many kinds of sensing devices for detection of CWAs have been reported,^1–3^ such as field-effect transistors, fluorescence, flame photometric, ion mobility spectrometry and gas chromatography in combination with mass spectrometry,^4–8^ but many of these instruments are bulky, expensive and require specific sample preparation as well as technically trained personnel. Due to the growing safety interest in environmental and working place, the detection of CWAs and their related compounds is more and more important today. Being as smart sensor system for the detection of trace toxic vapor, surface acoustic wave (SAW) microsensors have shown great promise as detectors for hazardous gases, especially for their high sensitivity, reliability, small size and low cost, short response time, and have been used to detected SO_2_,^9^ H_2_S,^10^ NO_2_,^11^ NH_3_,^12^ methane,^13^ hydrogen^14,15^ and explosives^16^ widely.

**Fig. 1:**
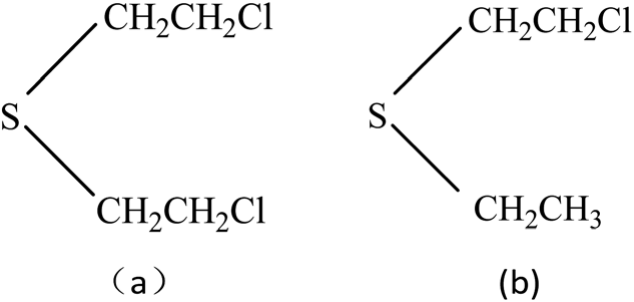
Chemical structures of mustard gas (a) and 2-chloroethyl ethyl sulfide (b).

As one of the most important CWAs, mustard gas, also named bis(2-chloroethyl) sulfide (HD), is a typical vesicant agent in chemical agent having been known, it has the title of “king of CWAs gas”. It not only can result in serious injury and possible death even at very low concentration because of its highly toxicity, but also has extensive damage to the animal and humanity. When the concentration of mustard gas in the air is 10 mg m^−3^, and when the concentration reaches 30 mg m^−3^, it can cause death within 2–5 minutes. The war leaves behind with terrorism create injury to people, and even death events are also obvious to all, so study on the sensor which detects mustard gas has reality and security significance. Since HD is highly toxic and its use is restricted in conventional laboratories, therefore, the research on HD is commonly conducted using simulant compound. Ideal simulant should mimic all the relevant chemical and physical properties of HD without their intrinsic toxicological properties. 2-Chloroethyel ethyl sulfide (CEES) is interesting as a suitable simulant for HD not only because of its similar chemical structure (–S–CH_2_CH_2_Cl, –S–CH_2_) with HD, but also depending on the physicochemical property which could be measured in detection experiments, Fig. 1. The use of SAW devices for detecting HD and CEES have been studied in some articles,^17,18^ as one kind of mass sensitive detector, the basic operating principle of SAW sensors must be the reversible adsorption of chemical vapor by adsorbent coatings which are sensitive and selective to the vapor being detected, so many films are selected as the coatings to detect HD and CEES previously, and the related mechanism of adsorption kinetics and the preparation of sensitive membrane materials have also been studied, such as ethyl cellulose (ECEL), poly(epichlorohydrin) (PECH), poly(ethylenimine), CdSnO_3_, *etc*, and PECH is considered to be the best film for the detection of HD or CEES, while the influencing factors including temperature, humidity, and interfering gases are seldom reported.^19–21^

However, SAW microsensor systems designed and fabricated to date have also shown several potential limitations, primarily involving chemical selectivity of the coatings and their reduced sensitivity due to temperature or humidity. The problems of sensitivity and selectivity of films depend on reversible interaction (*e.g.* solubility) with specific gas molecules, while the environment condition would give serious effect on the coatings, especially the temperature and humidity always play a very important role on SAW systems and cause undesirable instability of SAW sensors,^22^ small changes in temperature or humidity might have a larger effect on baseline stabilities than on the responses to the vapors.^23–26^ At same time, to have a reusable chemical sensor for real-time monitoring the environment, all the influencing factors should be considered in the kinetically sorption process. CEES is always used to be the simulant of HD and it is utmost important to develop the detectors to detect HD. As CEES contains a single chlorine atom on the β carbon relative to the sulfur atom (mustard is 2,2-dichlorodiethyl sulfide), but it is much less toxic, so it is expected that CEES could closely mimic the reactivity of HD. In this paper, polymer PECH, because of its strong hydrogen bond acidic chloroethyl functional group, was chosen as the sensitive film for the detection of CEES.^27,28^

## Analysis of adsorption theory in SAW gas sensor

**Fig. 2:**
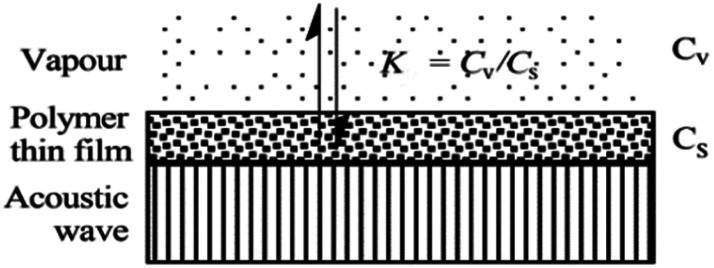
Reversible vapor absorption.

When SAW sensors are used, according to adsorption theory, chemical selectivity is interpreted in terms of the solubility properties of the vapors and polymers,^29,30^ the adsorption–desorption equilibrium can be expressed as *K* = *C*_s_/*C*_v_, where *K* is partition coefficient, *C*_s_ is the concentration of vapor in the sorbent phase, that is the polymer film, and *C*_v_ is the concentration of target gas in the vapor phase Fig. 2.

Then, the Linear Solvation Energy Relationship (LSERs) could be given as in eqn (1).1
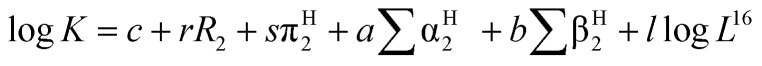
where, *R*_2_, π^H^_2_, 
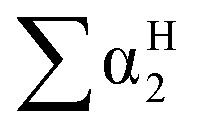
, 
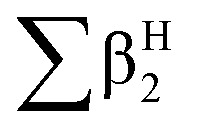
, log *L*^16^ are the vapor solvation parameters; coefficients *r*, *s*, *a*, *b*, and *l* are LSER coefficients related to each specific polymer; and *c* is the constant arising from the multiple linear regression method used to determine the LSER coefficients, the coefficients of PECH are shown as Table 1. From Table 1, the desired values of *s*, *a*, *b* and *l* coefficients are found, especially for the coefficient *b*, its big value is enough to indicate the hydrogen bond acidity, so PECH is considered to be the sensitive and selective film for SAW sensor.

**Table 1:** LESR regression coefficients for PECH

Polymer	Abbr.	Method	*c*	*r*	*s*	*a*	*b*	*l*	*R*	Std error
Poly(epichlorohydrin)	PECH	SAW	−0.75	0.44	1.44	1.49	1.3	0.55	0.993	0.11
Conclusion	Well-behaved polymer-coated SAW sensors									

## Materials and fabrication of SAW device

### Materials

2-Chloroethyl ethyl sulphide, poly(epichlorohydrin), toluene, and other reagents used in this study were AR grade and were purchased from Aladdin Chemical Reagent Company (Shanghai, China), pure N_2_ was provided by Beijing Haipu Company (Beijing, China), laboratory for temperature and humidity was built by our team, and frequency shifts were recorded by computer workstation.

### Fabrication of SAW device

**Fig. 3:**
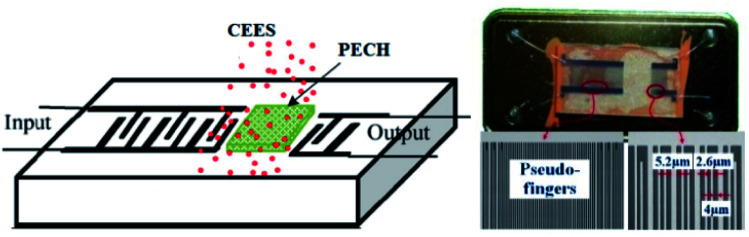
The schematic diagram (left) and the prepared SAW sensing device (right).

In this work, the SAW delay line pattern with two photolithographically defined aluminum (Al) transducers was fabricated on ST quartz wafer, and the two transducers mentioned above were separated by a path length of 2.5 mm. Single phase unidirectional transducers (SPUDTs) described in Fig. 3, confining the acoustic wave predominantly in one direction on the piezoelectric substrate surface, were used to form the transducers to reduce the insertion loss.^14^ Also, combed transducers were used to structure the left transducer to improve the oscillation stability by controlling the single mode selection. The sensing device was designed to operate at 150 MHz, and corresponding wavelength *λ* was ∼21 μm, and the electrode widths in SPUDTs were 5.2 μm and 2.6 μm, respectively. The lengths of the two transducers of the SAW devices were set to 260*λ* and 80*λ*, respectively. The fabrication procedure of the sensing device was depicted below. Aluminum with thickness of 200 nm was deposited on the cleaned ST quartz substrate using thermal evaporator. Then, a 1 mm-thick photoresist (PR) was spin-coated, exposed, and developed for the delay line patterns. Al was wet etched and PR was dissolved in acetone. After preparation of the Al electrodes, a 50 nm SiO_2_ thin-film was overlaid to the transducers to provide a good protect in process of PECH coating. Finally, the piezoelectric wafer with SAW device pattern was dicing-sawed for wafer bonding and packaging.

Then, the PECH was dissolved in chloroform, and dipped onto the developed SAW device surface, and dried with N_2_ at room temperature to build the sensing devices. The PECH film thickness could be estimated by monitoring the frequency shift.^31^ The optical and SEM picture of the sensing device was shown in Fig. 3, in which, the widths of the electrodes in SPUDTs are measured by ∼5.2 and 2.6 μm, and a spacing among them is 4 μm, agreeing well with the set parameters.

### Sensor measurement setup

**Fig. 4:**
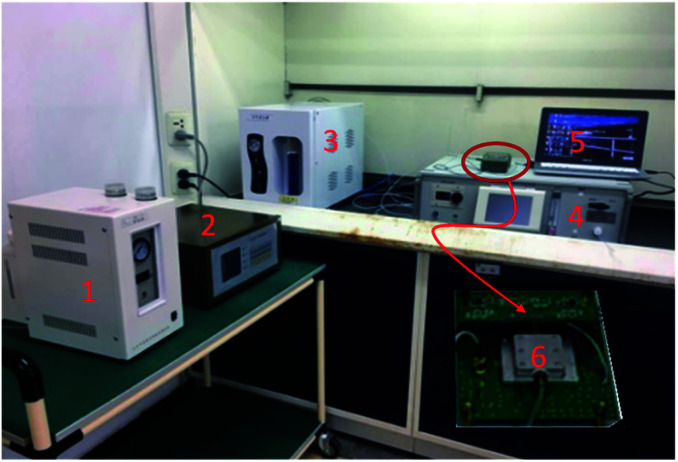
Sensor measurement system (1-hydrogen generator, 2-flame photometric detector, 3-air generator, 4-CEES generator, 5-PC, 6-prepared SAW sensor system).

The prepared sensing device and a naked device used as reference were placed in a surface nickel-plated Al gas chamber with volume of 500 ml, and connected into a differential oscillation loop to build the sensor system as shown in Fig. 4. The differential structure was used to eliminate the external temperature effect. The mixed oscillation frequency signal in kHz range was picked by a frequency counter, and connected by PC.

To obtain more realistic datum, room air was used as background gas. CEES was injected into the gas chamber in a generation system designed by our group depicted in Fig. 4. By blowing of N_2_ and diluting of room air, the given concentration of CEES gas was generated, and monitored by flame photometric detector (FPD) in real time. The generated CEES with various concentrations were exposed to the SAW sensing devices, and corresponding sensing signals towards CEES were collected and recorded by PC. The experiments towards environmental characteristics were conducted by varying the test temperature and humidity using temperature/humidity controlling system made by Siemens Co. with precision of ±0.5 °C and 1% RH. The smoke effect evaluation experiments were performed in a closed laboratory after burning some leaves and the smoke were in stable for an hour.

## Results and discussion

### Characterization of PECH film

**Fig. 5:**
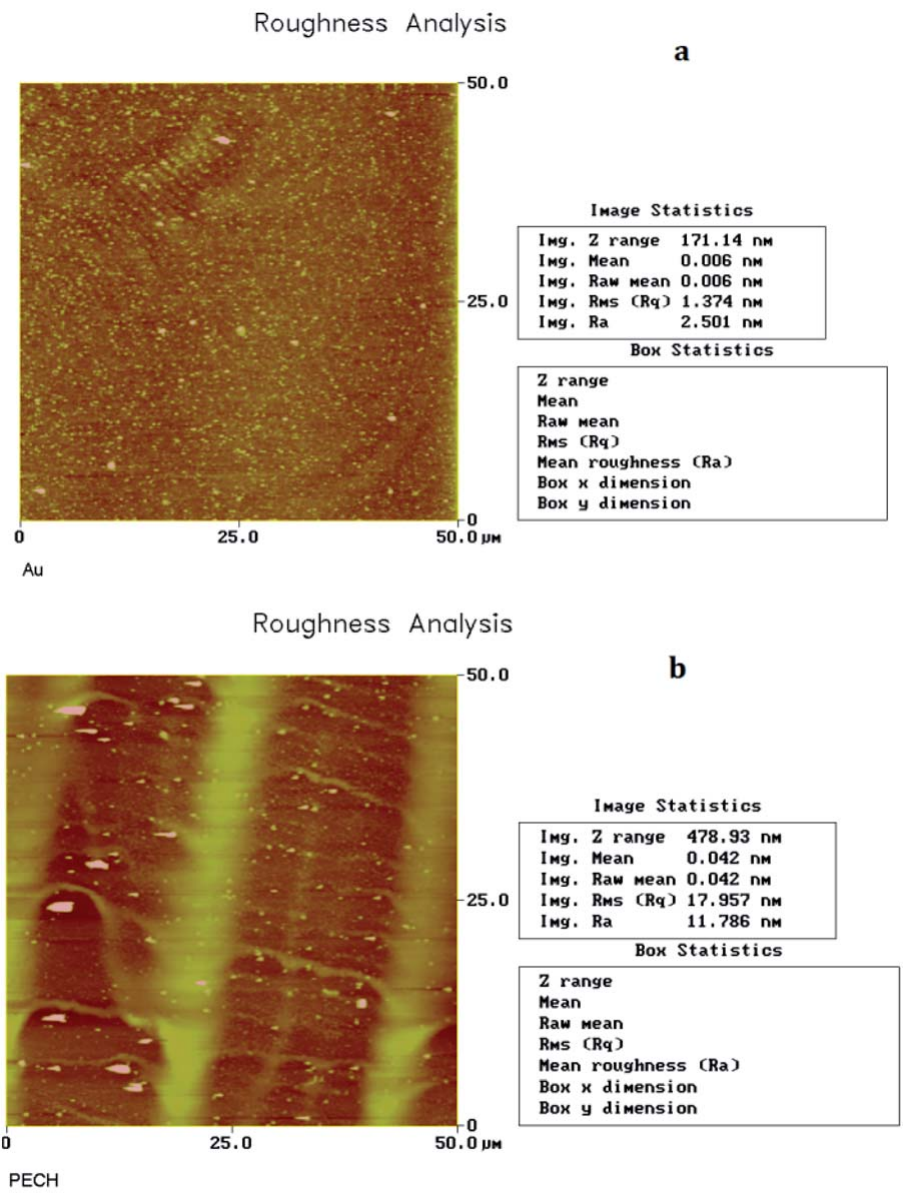
Roughness analysis of PECH uncoated (a) and coated (b) delay line. Resolution factor is 50 μm × 50 μm.

The scanning electron microscope (SEM) picture of the prepared PECH film was shown in Fig. 5. The uncoated surface (a) of sensing device was smooth and neat, while for the coated surface (b), because of the existence of PECH, the surface was rough and had no regular geometry, and porous surface from the amorphous PECH was benefit for the CEES adsorption.

### Sensitivity and repeatability testing

**Fig. 6:**
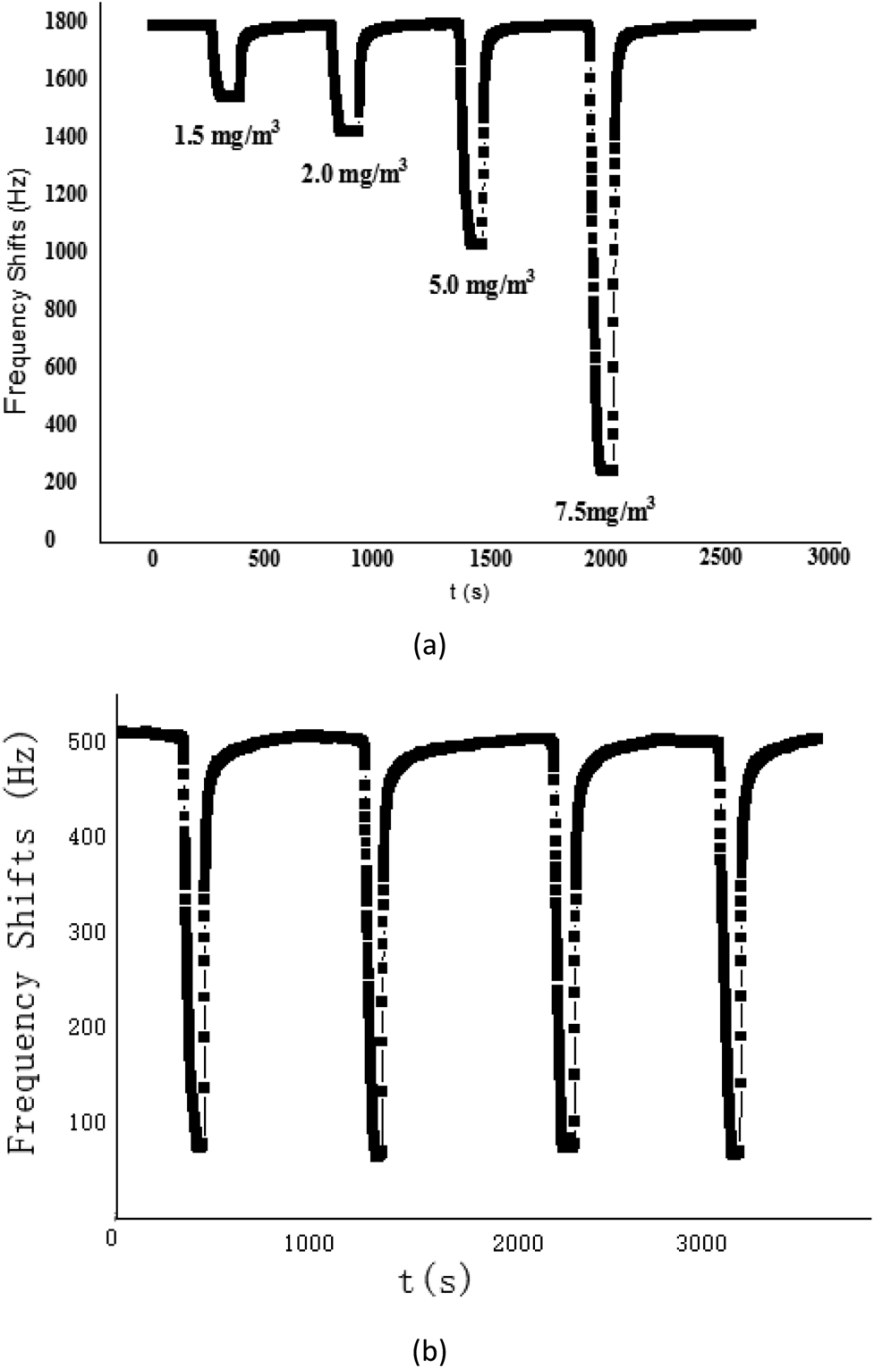
(a) The response of SAW-PECH sensor to different concentration of CEES; (b) repetition experiment of SAW-PECH sensor.

The proposed SAW sensing device was exposed to CEES with various concentrations to describe its sensitivity, as shown in Fig. 6(a). As the CEES concentration increased, the sensing response also increased, and it was quite linear for CEES at the concentrations of 1.2–10 mg m^−3^, the sensitivity defined by the fitted slope was evaluated as 233.17 Hz/(mg m^−3^). This plot indicated the specific, preferential vapor/oligomer interaction at low concentration, this interaction was likely to be hydrogen bond formation between hydrogen bond donating (HBD) groups on the PECH and hydrogen bond accepting (HBA) sulphur atoms in CEES, so the PECH polymer shows a high sensitivity to CEES.

To further verify the short term repeatability of the PECH-coated SAW sensor, four successive experiments were carried out, Fig. 6(b), it showed that the sensor was reproducible, we suggested that the proposed SAW gas sensor coated with PECH sensitive films was very promising for CEES gas detection application.

### Temperature effect on sensor response

Temperature not only affects the SAW velocity but also changes the physical dimension (and hence delay time) of the device, from theory, it is difficult to predict the specific dependence of a SAW vapor sensor on temperature. In general, from thermodynamic considerations, as the temperature increases, the vapor pressure, and thus the concentration of a gas in equilibrium with its adsorbed phase increases, the solubility of the vapor in a polymer would decrease, thereby reducing the response (and the sensitivity) of a polymer coated SAW sensor, so the effect of increasing temperature would be to decrease the concentration of dissolved vapor within the coating, but increase the rate of diffusion of the vapor molecules within the polymer.

The effect of temperature on the prepared SAW sensor responses was determined from 0 °C to 50 °C by CEES at the concentration of 8.0 mg m^−3^, the results were illustrated in Table 2. The values in the Table 2 clearly showed the trend that the responses of SAW sensor to CEES decreased with increasing temperature as expected, the sensitivity of various temperature were calculated at each experimental temperature as Hz m^3^ mg^−1^, and the sensitivity of a SAW sensor did indeed decrease with the increasing temperature. Even though there was reduced sensitivity at high temperatures, the rate of coating PECH response to CEES would increase due to the more rapid rate of diffusion, thus at higher temperatures the time required to attain maximum response would be shorter.

**Table 2:** The response value of SAW-PECH sensor to CEES at different temperature

Temperature (°C)	Frequency shifts (Hz)	Response time (s)	Recovery time (s)	Sensitivity (Hz m^3^ mg^−1^)
0	2757	70	15	551.5
10	1263	35	9	252.7
20	8180	19	8	162.2
30	431	9	7	64.8
40	198	9	7	35.4
50	122	12	7	24.6

**Fig. 7:**
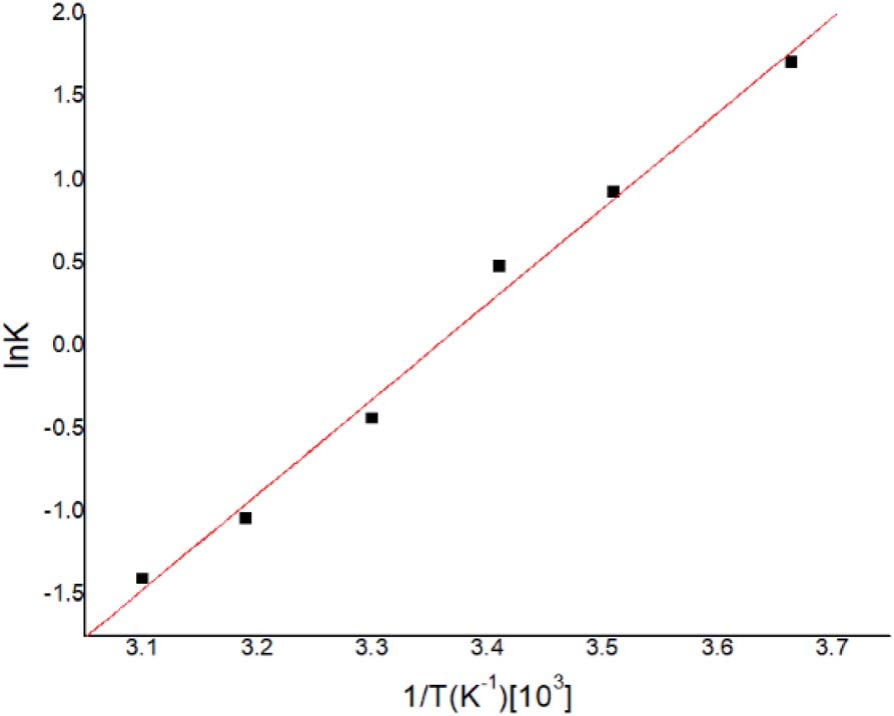
Temperature dependence for CEES sorption into PECH.

Gas–liquid chromatograph (GLC) studies have shown that the temperature dependence of *K* (partition coefficient) at low vapor concentration over finite temperature ranges can be described by the Arrhenius-type relationship.^32,33^2
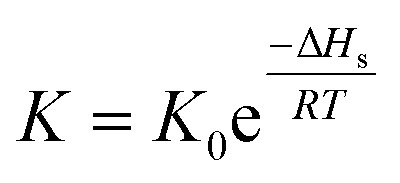
3
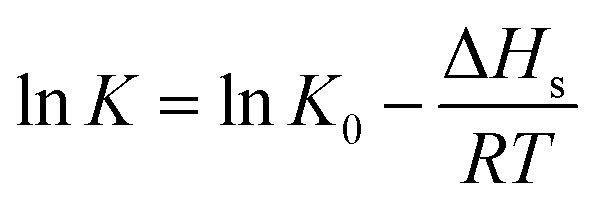
where the preexponential term *K*_0_ is, to a first approximation, independent of temperature, Δ*H*_s_ (KJ mol^−1^) is the heat of sorption, *R* (KJ mol^−1^ K^−1^) is the gas constant, *T* (K) is the absolute temperature, the results at 8.0 mg m^−3^ CEES were illustrated in Fig. 7, the linear relation between ln *K* and *T*^−1^ could be fitted as eqn (4).4
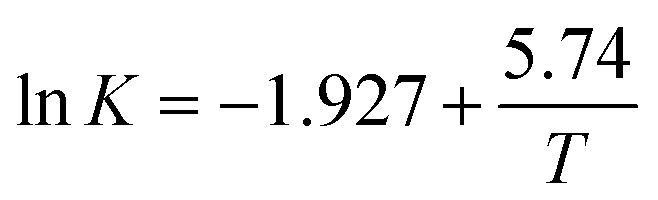


According to eqn (4), *K*_0_ and Δ*H*_s_ were calculated as 0.146 and −47.71 (KJ mol^−1^) respectively, and the results were very similar with previous reported work.^34,35^

### Humidity effect on sensor response

**Fig. 8:**
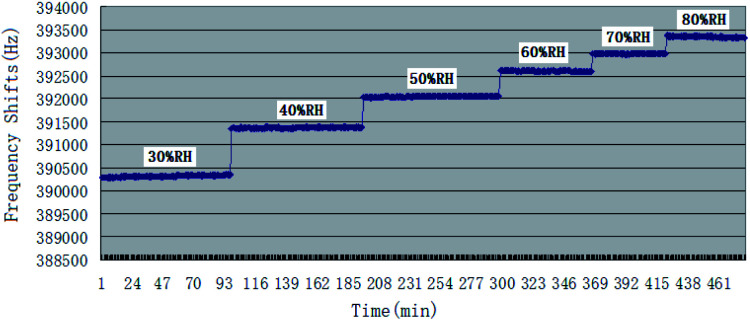
Baselines under different humidity for SAW sensor coated with PECH.

Being as one kind of mass-sensitive sensors, in SAW dual delay line, the uncoated reference channel is often used in effort to eliminate the influence of humidity, pressure or temperature, while the difference in the humidity behavior of the two delay lines still cause undesired instability. The controlling of humidity could be at least as important to the accuracy of measurements as the inherent selectivity and sensitivity for the target vapour, and many studies have been reported before about it.^36^ This paper examined the effect of humidity on the baseline frequencies and the responses to CEES of PECH coated SAW sensor. The response of sensor to atmospheric humidity was determined by exposure at 26 °C to air at RH values ranging from 30% RH to 80% RH, Fig. 8.

In Fig. 8, the base line of SAW sensor increased with the increasing of humidity, it could be explained as the increase of unit water molecular in PECH surface because of SAW mass-sensitive characteristics.

**Fig. 9:**
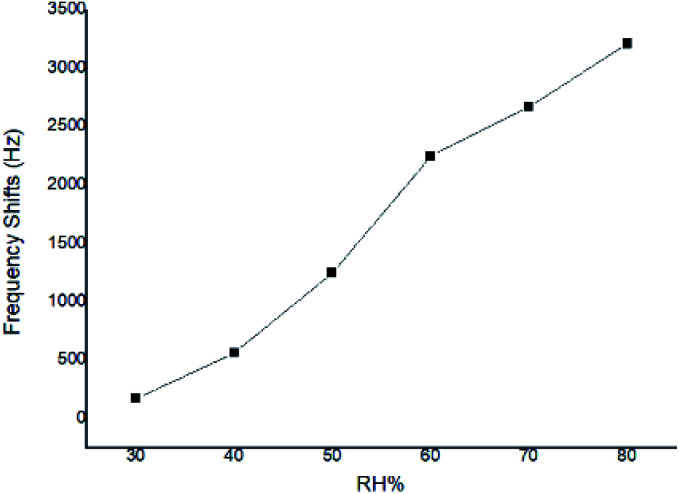
Responses of SAW sensor coated with PECH to CEES under different humidity.

At a given CEES concentration of 5.0 mg m^−3^, the effect of atmospheric humidity level on the responses to SAW sensor coated with PECH was examined in 30%, 40%, 50%, 60%, 70%, 80% RH, the results were showed in Fig. 9, where a higher response was observed for PECH coated sensor at the higher humidity levels. The reason might lie in the viscoelastic property of PECH, because when the relative humidity increased, there was more significant plasticization or solvation by CEES for polar PECH polymer, also there were more active sites between PECH film and CEES vapor to forming hydrogen bond (h-b), in this case, the presence of CEES vapor apparently leaded to a steady increase in vapor solubility in PECH, we speculated that the increasing of the humidity was at least partly conductive to the interactions between CEES vapor and PECH substrate.

### Interferent gases effect on sensor response

To further confirming the effectiveness of SAW sensor coated with PECH, many other gases whose concentrations were higher 100–1000 times than that of CEES were chosen and detected to carry on comparative experiments, the experimental results were shown in Table 3.

**Table 3:** Response of SAW sensor coated with PECH to interferential vapor (22 °C, RH = 35%)

Class	Interference gases	Concentration (mg m^−3^)	Frequency shifts (Hz)
Alkanes	*N*-Hexane	10 000	211
	Cyclohexane	10 000	505
	Heptane	10 000	462
Halogenated hydrocarbon	Trichloromethane	10 000	1542
	Carbon tetrachloride	10 000	867
Alcohols	Methanol	10 000	374
	Ethanol	10 000	353
	Isopropyl alcohol	10 000	1045
Aldehydes and ketones	Formaldehyde	10 000	909
	Acetone	10 000	460
	Ethyl acetate	10 000	1080
Ethers	Ethyl ether	10 000	160
	Petroleum ether	10 000	124
	Tetrahydrofuran	10 000	1285
Aromatic compounds	Benzene	10 000	793
	Methyl benzene	10 000	2920
Amine	Ammonia	10 000	888
	Aniline	10 000	40 540
	Ortho anisamine	2000	8963
Organophosphorus	Omethoate	1000	11 957
	Dichlorvos	1000	34 474
	Phoxim	1000	3539
	Parathion	1000	4020
	DMMP	1000	15 460
Organic acids	Formic acid	10 000	1731
	Acetic acid	10 000	5164
	Caproic acid	10 000	4252
Others	H_2_O	3000	2834
	Acetonitrile	10 000	1085
	90# gasoline	10 000	2017
	0# diesel oil	10 000	13 517

In general, common organic solvents and gases did not influence the performance of SAW sensor coated with PECH, organic amines, organophosphorus agrochemicals and organic acids would produce some influence because of their much higher concentrations, in higher concentration, the frequency shifted greatly, but their responses were obviously lower than that of CEES at the same concentration. Although the SAW sensor coated with PECH could response well to CEES, to some compounds whose structures are similar with CEES or HD, the exact structure of the compounds could not be recognized, and this result was entirely predictable, as the selectivity of a film depends upon reversible interactions (*e.g.*, solubility) with specific vapor molecules, it would be very unexpected to find a coating that would not interact to some limited extent at least with many potential interfering vapor, so SAW sensor arrays combined with pattern recognition or artificial intelligence might be used to solve this selectivity problem according to the differences of frequency shifts and response time.

### Smoke effect on sensor response

**Fig. 10:**
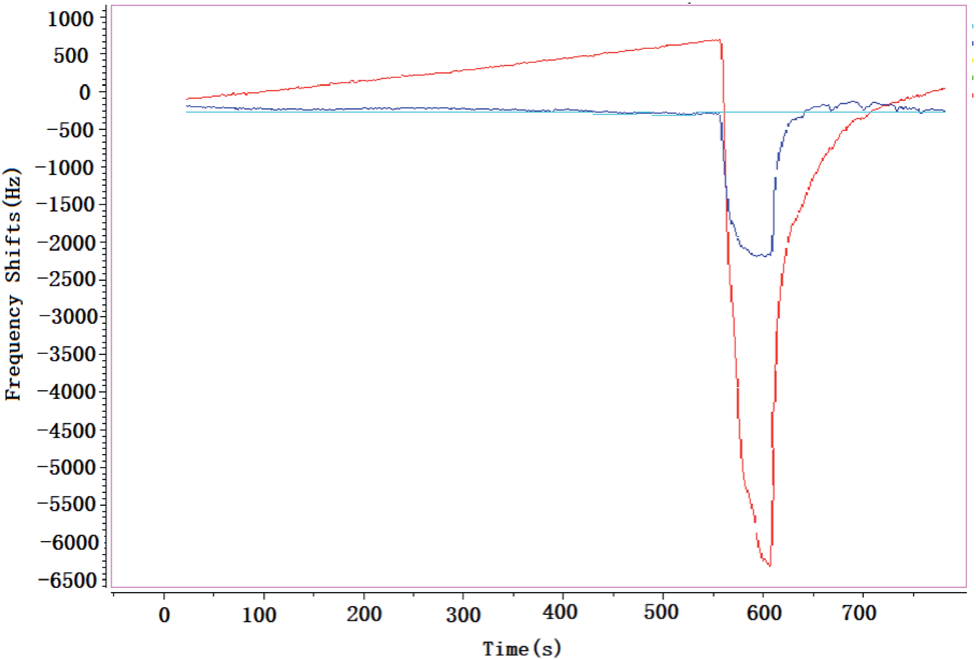
Influence of smoke on SAW sensor (22 °C, 36% RH).

Almost all the sensors or detectors with different principles are easily influenced by smoke, and it makes the target gases could not be detected. It is obvious that if the vapors have existed in smoke, it is almost impossible to detect them; but if the smoke is taken as background, and then the target gases are added in this smoke, the vapors could be detected easily. In this paper, a brief study was undertaken to verify influence of smoke on SAW sensor, Fig. 10.

When environment was full of smoke, CEES was detected at the concentrations of 3.0 mg m^−3^ (blue line) and 5.0 mg m^−3^ (red line) respectively, the absorption–desorption process were very obvious. That means that, no matter how complex the environment is, SAW sensor could always take it as background, in a sense, it is an advantage of SAW sensor compared with other technologies, and it might be more practical especially in the detection of CWAs that leaked into a complex environment suddenly.

### Aging studies

The results of the SAW sensor/coating aging studies were given in Table 4. At a given concentration of CEES 5.5 mg m^−3^, the SAW sensor was evaluated at regular interval months in 18 months. From the data in Table 4, even though some attenuation was observed in coating and sensor response over the duration of the tests, it was not so obvious, it was believed that the decrease of response was resulted in by the attenuation of PECH film and the variations in certain experimental variables from experiment to experiment.

**Table 4:** Aging studies of SAW sensor coated with PECH (25 °C, 35% RH)

Time (month)	0	1	3	6	12	18
Frequency (KHz)	0.915	0.8954	0.875	0.845	0.812	0.765
Attenuation (%)	0	2.19	4.37	7.65	11.26	16.39

**Fig. 11:**
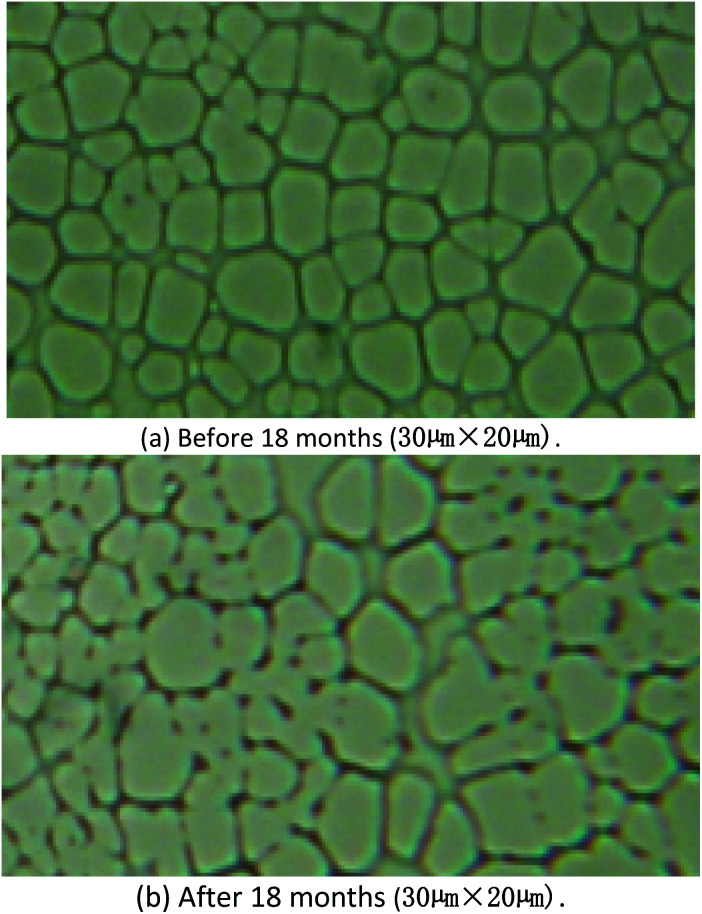
Image of PECH before (a) and after (b) 18 months storage.

From the image of optical microscope, it could be find the surface was almost same, Fig. 11. The results of aging studies indicated that the sensitivity of the SAW sensor coated with PECH was stable over a period of 18 months, and the SAW sensor would be retained for possible continuation of the future studies.

## Conclusions

In this work, PECH was prepared on the surface of SAW dual delay line successfully, and was selected to be as sensing layer for highly sensitive CEES detection, and the limit was about 1.5 mg m^−3^. It had been shown that the SAW sensor coated with PECH film decreased in vapor sensitivity with increasing temperature in the range from 0 °C to 50 °C due to the more rapid rate of diffusion and the decreasing solubility of CEES in PECH polymer. Being as the most important factors, responses of humidity from 30 to 80% RH were invested, and significant changes in sensitivity to CEES were observed as a function of atmospheric humidity for SAW sensor, in interferent gases, common organic solvents and gases would not interference the performance of the SAW sensor, the results of aging studies proved that the sensitivity of SAW sensor coated PECH film was stable during 18 months, and the larger question of long-term stability must be addressed in application.

## Conflicts of interest

The authors declare no conflict of interest.

## Supplementary Material
